# Detecting defects that reduce breakdown voltage using machine learning and optical profilometry

**DOI:** 10.1038/s41598-024-57875-5

**Published:** 2024-03-28

**Authors:** James C. Gallagher, Michael A. Mastro, Alan G. Jacobs, Robert. J. Kaplar, Karl D. Hobart, Travis J. Anderson

**Affiliations:** 1 U.S. Naval Research Laboratory, 4555 Overlook Ave SW, Washington, DC 20375 USA; 2 Sandia National Laboratories, 1515 Eubank Blvd SE, Albuquerque, NM 87123 USA

**Keywords:** GaN, Vertical diodes, III–V semiconductors, Machine learning, Optical profilometry, Electronic devices, Scientific data

## Abstract

Semiconductor wafer manufacturing relies on the precise control of various performance metrics to ensure the quality and reliability of integrated circuits. In particular, GaN has properties that are advantageous for high voltage and high frequency power devices; however, defects in the substrate growth and manufacturing are preventing vertical devices from performing optimally. This paper explores the application of machine learning techniques utilizing data obtained from optical profilometry as input variables to predict the probability of a wafer meeting performance metrics, specifically the breakdown voltage (V_bk_). By incorporating machine learning techniques, it is possible to reliably predict performance metrics that cause devices to fail at low voltage. For diodes that fail at a higher (but still below theoretical) breakdown voltage, alternative inspection methods or a combination of several experimental techniques may be necessary.

## Introduction

There has been a growing interest in using machine learning (ML) for semiconductor device design and optimization in recent years due to the availability of larger datasets generated by semiconductor foundries^1^. However, due to the massive amount of data required to train these models, ML’s uses are limited for less mature semiconductor material systems. Wide bandgap semiconductors fall into this category. Furthermore, in the GaN and Ga_2_O_3_ material systems the substrate manufacturing and epitaxial growth processes are yet to be optimized, and the defects causing them to behave sub-optimally are often unknown.

Even with the limited datasets available to date, utility in studying wide bandgap semiconducting datasets with ML has been reported. Success has been demonstrated using computationally generated datasets from TCAD simulations^2,3^, which can predict CV and IV behavior of Ga_2_O_3_ Schottky Barrier Diodes^4–6^. Some experimental ML models have been used to predict transistor current and switching voltage from gate currents and input voltages^7^. Models have been trained to classify the constituent chemical compounds of a semiconductor by using photoluminescence^8^. ML has also been used to predict the quality of GaN Ohmic contacts from the fabrication recipe^9^. Previous work has shown that we can predict low voltage properties of vertical GaN diodes with over 75% accuracy from optical profilometry data using both convolutional neural networks^10^ and simpler models^11^ (logistic regression^12^, decision tree^13^, and K nearest neighbor^14^ (KNN)). This work expands on this by using high voltage breakdown as the quality metric and discusses the possible defects causing reduced diode performance.

Different defects do not have an equal effect on the semiconducting properties and devices’ performance. In SiC, for example, it was found that basal plane dislocations significantly impact vertical devices while threading edge dislocations are benign^15^. For GaN devices, the cause of electrical performance drop is not always known, though there has been some successful research in identifying degradation and failure mechanisms. It has been shown that non-homogeneous conductivity can degrade performance of vertical GaN devices^16^, the diffusion of Mg and H into the active region of GaN LEDs can degrade performance^17^, and threading dislocations have been shown to increase leakage current, reduce breakdown, increase ON resistance, and reduce the switching frequency^18^. Many types of defects affect the crystal structure in GaN, with a significant proportion discernable via observable morphological defects on the surface and small defects in the substrate that often extend through the epitaxial layers during growth. Hence, optical profilometry provides a data-rich input for developing ML models for wafer screening^11,19–22^. This study involved the collection of optical profilometry data from seven GaN PiN diode wafers by employing a combination of data pre-processing and supervised machine learning algorithms to develop predictive models for different targeted performance metrics.

## Experimental details

**Figure 1:**
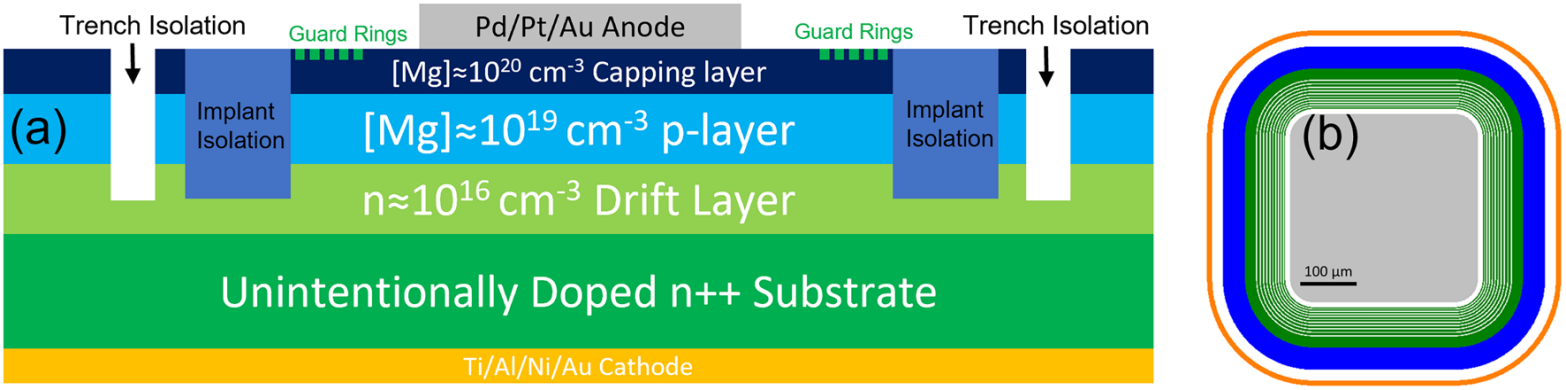
A side view (**a**) and a to-scale top view (**b**) of the diode design are shown. The displayed figure represents a device with anode area of 0.116 × 10^–2^ cm^−2^ though areas ranging from 0.0911 × 10^–2^ to 0.560 × 10^–2^ cm^−2^ are included in this study.

GaN was grown homoepitaxially using MOCVD in a Taiyo Nippon Sanso SR4000HT reactor at atmospheric pressure. The epitaxial structure consisted of the following layers: 8 µm Si-doped (n ~ 10^16^ cm^−3^) layer, 500 nm p-GaN layer ([Mg] ~ 10^18^–10^19^ cm^−3^), and 15 nm capping layer ([Mg] > 10^20^ cm^−3^) (see Fig. 1 for diagram). The sample was annealed to remove hydrogen, as hydrogen complexes with magnesium are known to prevent p-layer conductivity.

To obtain input data for the model, optical profilometry was used to create a 3D image of the surface of the wafer before any lithography steps were performed. Measurements were taken on a 2-inch wafer using a Zygo™ NewView 7300 optical profilometer with a 2.5 × magnification, giving an x–y resolution of 4.4 µm/pixel. Several images were stitched together to map the full wafer, using Zygo's stitching algorithm in their MetroPro software with 25% image overlap to minimize stitching artifacts.

The input data for this program consisted of four parameters: the root mean squared (RMS) roughness, the number of bumps and pits, the size of the devices, and the distance from the center of the wafer. The first two parameters are extracted from optical profilometry in the same manner as they were in our previous research^11,23^. The RMS roughness was extracted using the standard $RMS=\sqrt{\frac{1}{N}\sum_{i=1}^{N}{\left({Z}_{i}-{Z}_{avg}\right)}^{2}}$, and the number of bumps and pits (called outliers in this work) were counted by determining which points fell outside of a Gaussian distribution using a generalized ESD function^24^. On all wafers these calculations were done on 325 µm × 325 µm squares with all data within 2 mm of the diode considered in the machine learning models, as discussed later.

Across the seven wafers, 3912 PiN diodes were fabricated using the following process (see Fig. 1 for diagram): The p-layer was isolated by N implantation. A guard ring structure was fabricated using the design optimized in our previous research^11,25,26^. An outer trench isolation layer was produced using Cl_2_ etching. Pd (20 nm)/Pt (10 nm)/Au (80 nm) metal contact pads were used as a topside contact. Ti (20 nm)/Al (120 nm)/Ni (40 nm)/Au (80 nm) was used as a backside contact.

**Figure 2:**
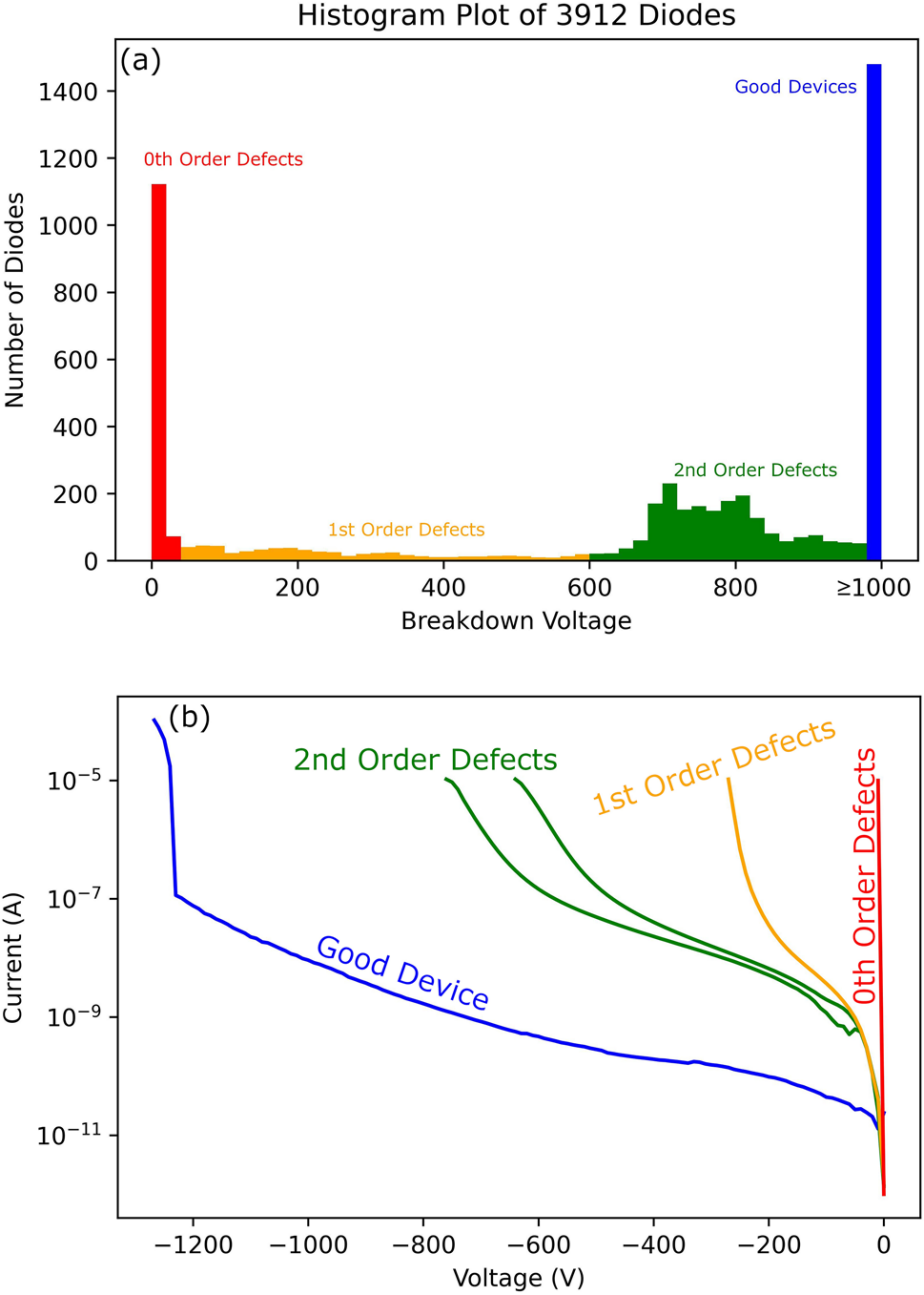
(**a**) A histogram plot showing the distribution of breakdown voltages for all 3912 diodes in this work. Using the breakdown voltage, the diodes were divided into four categories related to the type of defects present: 0th, 1st, 2nd, and only higher order defects (Good Devices). (**b**) Example curves from the histogram plot in (**a**).

To confirm the diode-like behavior, low voltage (− 10 to 10 V) IV sweeps were performed using a Keithley 4200-SCS semiconductor parametric analyzer to ensure that the diodes blocked in reverse bias and turned on in forward bias. This behavior was observed in > 99% of the diodes. High voltage breakdown measurements were taken with the Keithley 237 or 2657A High Power System Source Meter to 1000 V in reverse bias, with the distribution of breakdown voltage shown in Fig. 2a. Some of the diodes that did not break down before 1000 V were measured at higher voltages in a vacuum system, and avalanching breakdown behavior was verified to match the theoretically expected breakdown voltage^25,26^.

## Results and discussion

For every diode, the breakdown voltage was measured at the datum where the reverse leakage current was about 1 µA. From the histogram plot showing the distribution of diode data in Fig. 2a, the defects causing a drop in breakdown can be classified into four categories: 0th order defects which cause catastrophic failures at low voltages, 1st order defects which greatly diminish the performance, 2nd order defects which mildly diminish the performance, and higher order defects which have no noticeable effect on breakdown and will not be discussed in this paper, though they may affect other properties of the diodes such as long term reliability. Example curves showing the reverse bias behavior with different defect orders are shown in Fig. 2b. Though the boundaries between the defect orders may be arbitrary for this study, we will set them as: V_Bk_ < 50 V contains a 0th order defect, 50 V ≤ V_Bk_ < 600 V contains a 1st order defect, and 600 ≤ V_Bk_ < 1000 V contains a 2nd order defect. These classifications were selected based on regions with large clusters of these defects. The 0th order defects have a clustering close to V_Bk_ = 10 V breakdown. 2nd order defects are clustered around V_Bk_ = 800 V breakdown, with 1st order defects occurring in the large gap between these numbers, and the “good device” category representing the large number of defects with breakdown above 1000 V. For each wafer screening technique, it is important to understand the types of defects it can detect and to which category they belong.

**Figure 3:**
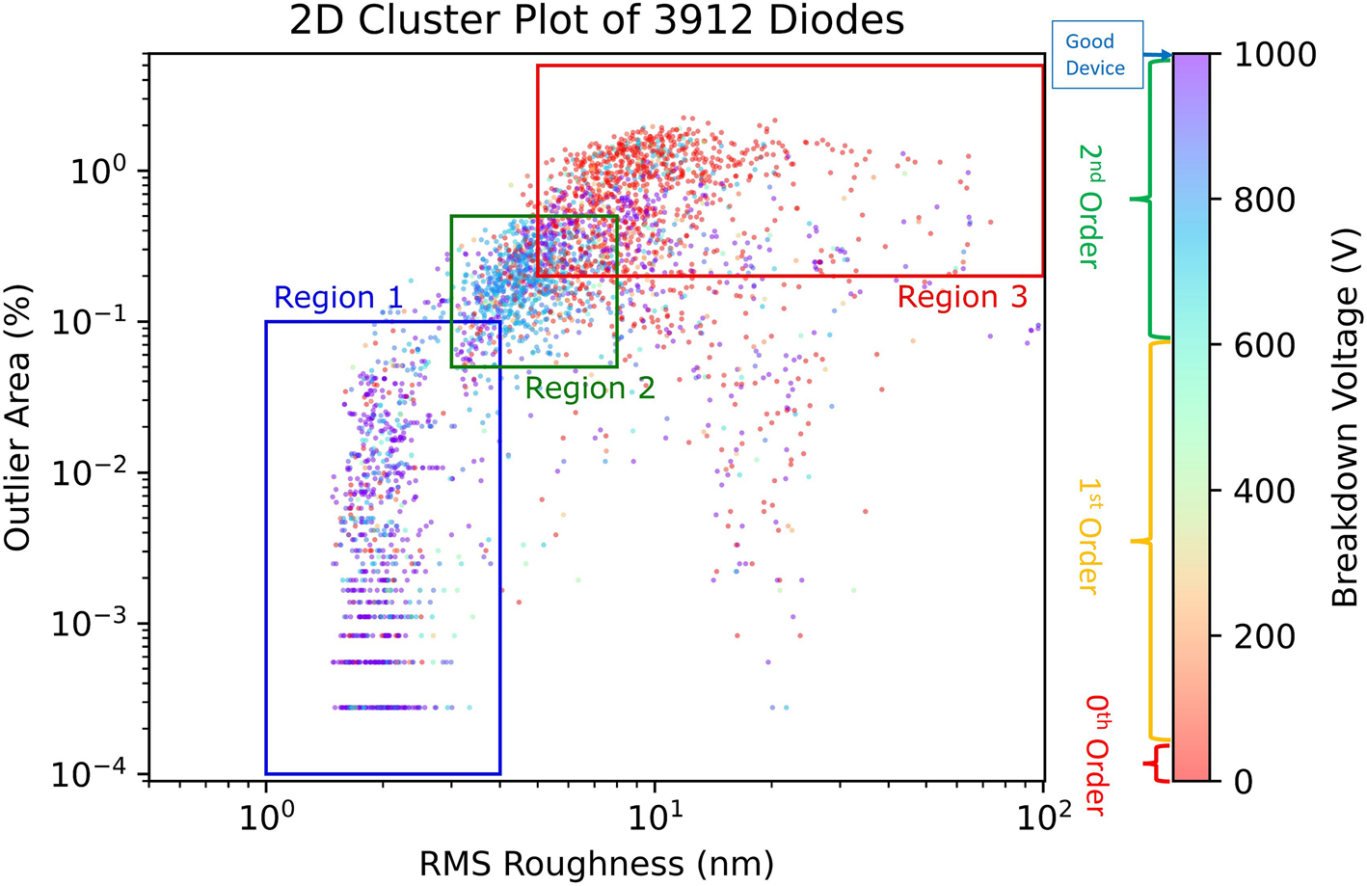
Cluster plot showing the relationship between the outliers (bumps and pits) and the RMS roughness from the optical profilometer related to the breakdown voltage of the diode. Three regions are highlighted on the plot: Region 1 contains the highest concentration of devices with correct current–voltage response, and Region 3 contains the highest concentration of devices with higher order defects. The number of devices containing each level of defect in each region is summarized in Table 1.

To correlate the diode performance with optical profilometry, a 2D cluster plot was constructed for the variables extracted from optical profilometry with the breakdown voltage (see Fig. 3). From this cluster plot, three regions containing at least 1000 devices are highlighted: Devices in Region 1 have a few outliers and a low RMS, Region 2 contains a moderate number of outliers and a higher RMS, while region three is very rough with many outlier points. Comparing the breakdown voltage across the three regions (see Table 1), there is a strong correlation between the odds of a 0th or 1st order defect occurring and the roughness of the region, as about 90% of the devices in this region do not contain a 0th or 1st order defect in Region 1, with the probability of a 0th or 1st order defect occurring increasing by 4× and 2× respectively in Region 2. In Region three the odds of a 0th order defect are the greatest, while the probability of a 1st order defect is consistent with that of Region 2. As for 2nd order defects, the results are less conclusive as the correlation is much weaker. There is a small but noticeable increase in probability moving from Region 1 to Region 2. Though there appears to be a drop moving to Region 3, it should be noted that a 0th or 1st order defect would overshadow a 2nd order defect leading to the illusion of a drop. The observed correlation indicates that the outliers detected, and the RMS are good variables to include in a machine learning model predicting the quality of diodes. It's important to note that device size and distance from the center are also important variables, with decision trees models frequently split based on these parameters at the second level. This probably because smaller diodes are less likely to encounter critical defects due to their reduced size, and defects are more commonly found towards the wafer's edge. However, it was rare that decision tree models split on these as the root node and the models would likely still be accurate without them as due to the strong correlation between breakdown voltage and optical profilometry parameters (RMS and number of outliers) shown in Fig. 3.

**Table 1:** The number of defects of each order in the regions shown in Fig. 3: 0th order V_Bk_ < 50 V, 1st order V_Bk_ 50–600 V, 2nd order V_Bk_ 600–1000 V, and good devices with only higher order defects (V_Bk_ ≥ 1000 V).

Defect order	Region 1		Region 2		Region 3	
0th order	78	5.3%	239	20.5%	311	35.3%
1st order	71	4.8%	134	11.5%	117	13.3%
2nd order	592	40.3%	625	53.7%	364	41.4%
Good device	727	49.5%	166	14.3%	88	10.0%

**Figure 4:**
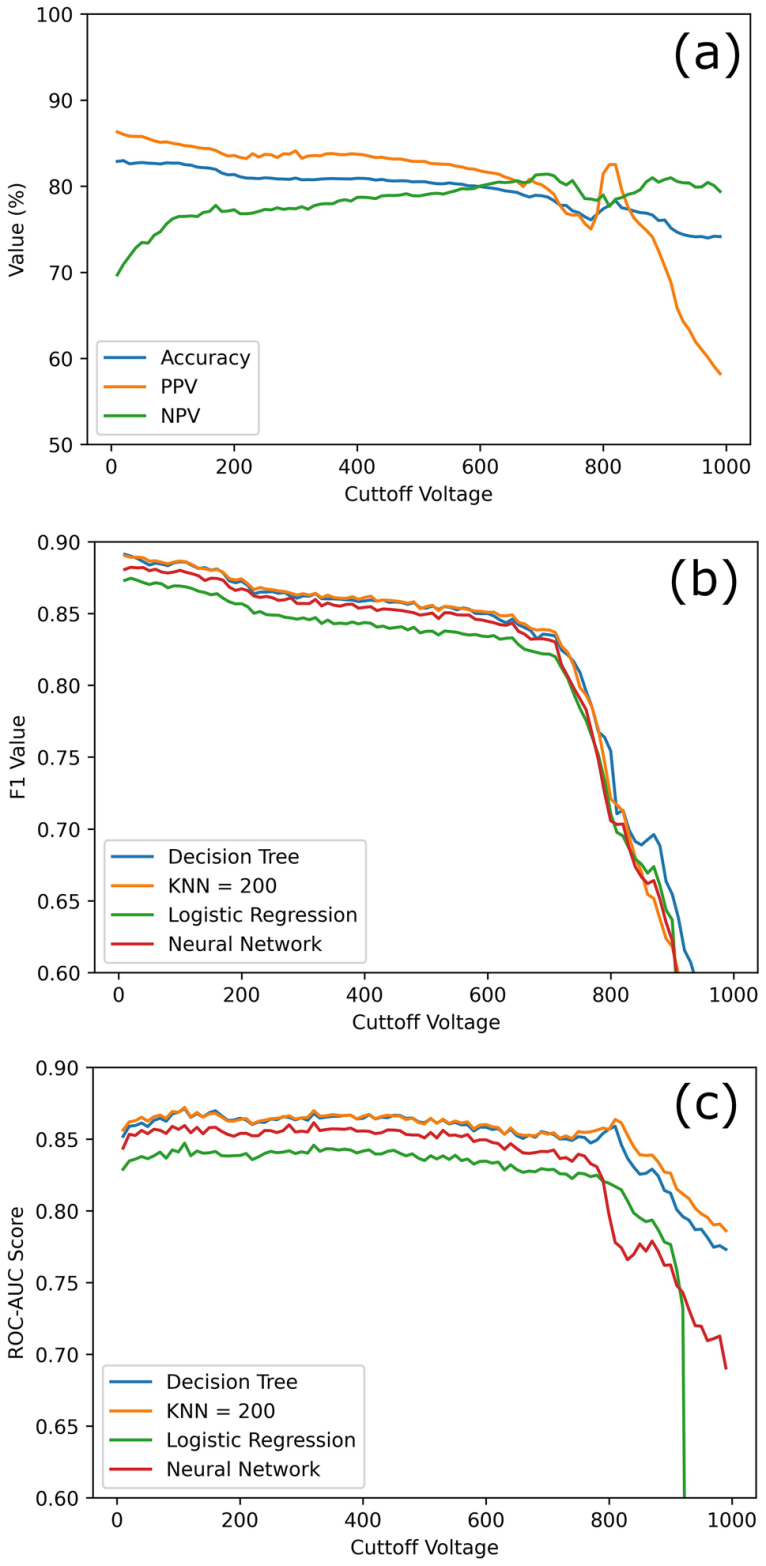
The three plots depict the prediction accuracy, including the Positive Predictive Value (PPV) and Negative Predictive Value (NPV) of the best model at each point (**a**), F1 value (**b**), and ROC-AUC Score (**c**), show the quality of the machine learning models vs cutoff voltage. A noticeable drop in quality occurs around a 700 V cutoff value.

The data were analyzed with machine learning using the four aforementioned input variables and V_Bk_ as the output variable. Four machine learning models were used: Decision Tree, K-Nearest Neighbors (KNN), Logistic Regression, and 2 Layer Neural Network with 2 neurons per layer. The models were optimized using a hyperparameter search^27^. The number of devices used in this study (3912) is too low to do a regression analysis to predict the exact breakdown voltage, so a binary pass/fail cutoff point was used instead. To explore the validity of each cutoff point, several possible values were tested, and the effectiveness of each model was evaluated using three parameters: the accuracy of the model (Fig. 4a), the F1 value (Fig. 4b), and the area under the receiver operating characteristic curve (ROC-AUC) (Fig. 4c). These values were extracted by training the four machine learning models on 80% of the data chosen randomly. The remaining 20% of the data was used to test the accuracy of the models. This was performed over 100 times at each cutoff voltage with new random sorting of the train and test data each time. The average values are reported in Fig. 4. The accuracy was determined by measuring the probability of a True Positive (TP) (V_Bk_ > Cutoff correctly predicted) or True Negative (TN) (V_Bk_ ≤ Cutoff correctly predicted) occurring. To ensure the model could predict both positive and negative values well, the Positive Predictive Value ($PPV=TP/(TP+FP)$) and the Negative Predictive Value ($NPV=TN/(TN+FN)$) with FP and FN being False Positives (V_Bk_ > Cutoff falsely predicted) and False Negatives (V_Bk_ ≤ Cutoff falsely predicted) were plotted along with the accuracy in Fig. 4a. The plot shows that although the accuracy is fairly consistent with a chosen cutoff value, there is a high FP rate when the cutoff > 850 V and a high FN rate when the cutoff < 150 V. A good cutoff value appears to be around 600 V as the Accuracy, PPV, and NPV are all at least 80% at that point. This is around the boundary used in this study to classify diodes as having a 0th or 1st order defect but not a 2nd order defect. The F1 and the ROC-AUC values are ML metrics for measuring the ability of the models to sort values. For F1 values, about 0.7 is often considered good with 1 being perfect prediction and 0 being no better than random. The ROC-AUC values of 0.8 or higher means the model is excellent at sorting in binary prediction methods, with 0.5 being no better than random^28^. These values are both excellent at cutoffs < 700 V, although a substantial decrease begins once a significant percentage of devices with only 2nd order defects are classified as passing. At low cutoff voltages, these values are still high despite the low NPV because it is compensated by a high PPV.

**Figure 5:**
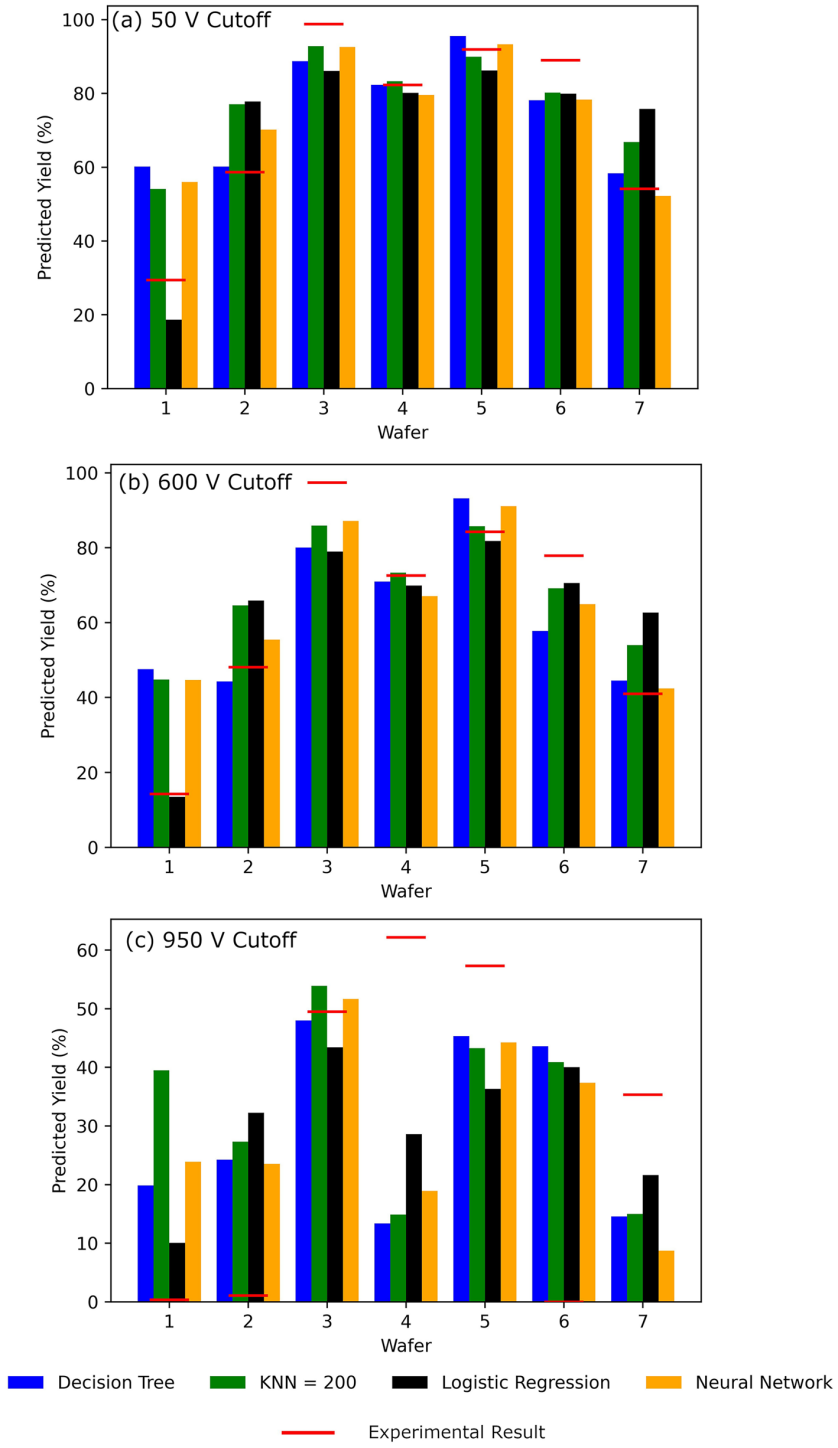
The experimental vs ML model yield for all seven wafers in this study is show for pass/fail cutoff of 50 V (**a**), 600 V (**b**), and 950 V (**c**) representing 0th, 1st, and 2nd order defect detection. The RMS error for all four models at all cutoffs is shown in Table 2.

Since the main objective of the ML models is to screen wafers, it is important to test the accuracy of this model at predicting the characteristics of a new wafer in the series. To test this, the ML models were trained using 6 of the 7 wafers, and the remaining wafer was used as a test. The yield was predicted by averaging the probability of a device having V_Bk_ > cutoff. This was tested three times at cutoff voltages of 50 V, 600 V, and 950 V breakdown shown in Fig. 5a–c. As an error measurement with the method, the RMS error = $\sum_{i}\sqrt{({Y}_{Exp\_i}-{Y}_{Pred\_i}{)}^{2}}/N$ was calculated with ${Y}_{Exp\_i}$ being the experimental yield, ${Y}_{Pred\_i}$ being the ML predicted yield, and *N* being the number of wafers = 7. From the results in Table 2, similar errors are shown with a 50 V and 600 V cutoff with the choice of ML model having little impact, but the value more than doubles when the cutoff voltage is set to 950 V. Additionally, when choosing a 50 V cutoff or 600 V cutoff for the breakdown voltage, the four ML models usually predict similar yields, which is close to the experimental result in 6 of the 7 wafers. The exception to this rule is wafer 1; however, it is possible that errors in the fabrication reduced the experimental yield causing a large overprediction in yield. When choosing a 950 V cutoff, only 1 of the 7 wafers is accurately predicted.

**Table 2:** The RMS of predicting the yield of error of the four ML models used in this study with cutoffs at 50 V (0th order defects fail), 600 V (0th and 1st order defects fail), and 950 V (all defects fail).

ML model	50 V	600 V	950 V
Decision tree	13.09%	16.58%	28.71%
KNN	13.24%	15.03%	31.07%
Logistic regression	13.28%	13.07%	25.24%
Neural network	11.72%	13.29%	30.31%

Experimental results are shown in Fig. 5

The results from this paper provide insights into the types of defects responsible for a reduction in breakdown. This study used primarily optical profilometry results as input variables, as it is effective at detecting extended defects. Defects that cause a large drop in breakdown voltage likely extend though large portions of the drift layer and the p-layer; therefore, the 0th and 1st order defects could be caused by imperfect surface morphologies on the substrate which propagate through the epitaxial layers during MOCVD growth. The 2nd order defects are more likely to be point defects or short-range extended defects as they can cause a smaller drop in breakdown by reducing electron mobility and creating a non-uniform electric field in the epilayers but won’t likely extend through the samples and won’t be detected on the surface. This illustrates why the machine learning models do well when detecting 0th and 1st order defects but falter with 2nd order defects. Incorporating input variables from spectroscopic and x-ray techniques is anticipated to enhance the model's capability to detect 2nd order defects as well.

## Conclusion

In conclusion, GaN PiN wafers were successfully fabricated using MOCVD, and PiN diodes were created following an optical profilometry study that was employed to comprehensively map the RMS roughness and identify the locations of bumps and pits, which are considered outliers as they deviate from a Gaussian distribution. High voltage reverse bias measurements of the breakdown voltage have demonstrated that the diodes can be classified into four distinct categories. These categories encompass the presence of 0th order defects, which may lead to catastrophic failures, as well as 1st order defects that result in a significant reduction in breakdown performance. Additionally, the study identified 2nd order defects associated with a milder decrease in breakdown and, intriguingly, the possibility of well-functioning devices with potential higher order defects, though these extend beyond the scope of this investigation. Correlating the optical profilometry data with breakdown voltage revealed a pronounced association between rougher samples featuring more bumps and pits and the formation of 0th and 1st order defects, while a potential, albeit weaker, correlation was observed with 2nd order defects.

Furthermore, the integration of machine learning models showcased their efficacy in predicting the probability of diodes being afflicted by 0th or 1st order defects with a high accuracy exceeding 80%. These models, with their ability to forecast wafer yields with a success rate of 6 out of 7 instances, promise to be a valuable asset for the industry. However, it is worth noting that their performance exhibited limitations in predicting 2nd order defects, suggesting they are likely caused by shorter range defects in the epilayers.

All acronyms and variable names used in this paper are listed in Table 3.

**Table 3:** Variables and acronyms used in this paper.

Acronym	Full form
ML	Machine learning
TCAD	Technology computer-aided design
CV	Capacitance–voltage
IV	Current–voltage
MOCVD	Metal–organic chemical vapor deposition
RMS	Root mean square
ESD	Generalized extreme studentized deviate (for outlier detection)
LED	Light emitting diode
KNN	K-nearest neighbors
PPV	Positive predictive value
NPV	Negative predictive value
ROC-AUC	Receiver operating characteristic-area under curve
PiN	P-type/Intrinsic/N-type (semiconductor layers)
V_Bk_	breakdown voltage
TP	true positive
TN	True negative
FP	False positive
FN	False negative
F1	F1 score (harmonic mean of precision and recall)

### Supplementary Information

Supplementary Information.

## Data Availability

The data used to train the machine learning models is available in the supplementary materials. The authors affirm the information needed to reproduce this work is available in the published article.
